# Limbic-visual attenuation to crying faces underlies neglectful mothering

**DOI:** 10.1038/s41598-019-42908-1

**Published:** 2019-04-23

**Authors:** Inmaculada León, María José Rodrigo, Wael El-Deredy, Cristián Modroño, Juan Andrés Hernández-Cabrera, Ileana Quiñones

**Affiliations:** 1 Instituto Universitario de Neurociencia, La Laguna, Canary Islands, Spain; 2 Facultad de Psicología, Universidad de La Laguna, La Laguna, Canary Islands, Spain; 3 Centro de Investigación y Desarrollo en Ingeniería en Salud, Universidad de Valparaíso, Valparaíso, Chile; 4 Departamento de Ciencias Médicas Básicas, Facultad de Ciencias de la Salud, Universidad de La Laguna, La Laguna, Canary Islands, Spain; 5 Basque Center on Cognition Brain and Language, Donostia-San Sebastián, Basque Country, Spain

**Keywords:** Neuroscience, Emotion

## Abstract

Neglectful mothering is one of the most common forms of childhood maltreatment, involving a severe disregard of the child’s needs, yet little is known about its neural substrate. A child’s needs are usually conveyed by signals of distress revealed by crying faces. We tested whether infant and adult crying faces are processed differently in two sociodemographically similar groups of Neglectful (NM) and non-neglectful Control Mothers (CM). We used functional brain imaging to analyze the BOLD response from 43 mothers (23 neglectful and 20 control) while viewing faces from infants and adults (crying and neutral). In NM as compared to CM, the BOLD responses to both infant and adult crying faces were significantly reduced in the cerebellum, lingual, fusiform, amygdala, hippocampus, parahippocampus, and inferior frontal gyrus. The reduced BOLD was also modulated by comorbid psychiatric symptoms. In the CM, frontal activation to infant versus adult crying faces was enhanced, whereas in the NM activation in the anterior cingulate cortex to infant crying was reduced compared to adult crying. The altered neural response to crying faces in NM, showing generic face and infant-specific face processing deficits, could underlie their characteristic poor social abilities as well as their poor response to infant needs, both affecting the caregiving role.

## Introduction

A substantial body of research has demonstrated the cumulative behavioral and neurobiological consequences of childhood maltreatment from infancy to adulthood^1–4^. Specifically, neglectful mothering, which involves a drastic disregard of the infant’s basic needs for food, clothing, shelter, medical care, supervision or emotional support is one of the most common forms of childhood maltreatment^5^. Of the children who experienced maltreatment in the U.S. population, 74,4% suffered neglect, 17.2% physical abuse, and 8.4% sexual abuse^6^. This neglectful pattern is known to be associated with a child’s insecure attachment to the caregiver, which in turn can be predictive of poor developmental outcomes and greater psychiatric vulnerability^5^. Despite its relevance, we are only beginning to elucidate the neural correlates of neglectful mothering. Knowing more about brain alterations in those mothers could improve our understanding of the neglectful behavior and potentially take a preventive stance or devise direct interventions.

Neglectful mothers (NM) often disregard the child’s needs to an extreme. Such needs are usually conveyed by signals of distress displayed in crying faces. Altered neural processing of emotional faces could explain their neglectful behavior. Electrophysiological analysis of the face-specific N170 signal showed that NM did not exhibit the expected increase associated with crying compared to laughing and neutral faces, indicating brain alteration in the early differentiation of crying infant faces^7^. Furthermore, in an analysis of structural connectivity, NM showed a reduced volume of anatomical tract interconnecting the face-responsive cortex and the limbic system, the right inferior longitudinal fasciculus (ILF), that was predictive of poor mother-child bonding interactions^8^. The maternal brain may also change as a function of early life experiences. Mothers who reported receiving higher maternal care in childhood exhibited higher activations in response to infant cries in the middle frontal gyrus, superior temporal gyrus, and fusiform gyrus, whereas mothers reporting lower maternal care showed increased hippocampal activations^9^. Extreme early life experiences such as a history of abuse and early deprivation were associated with increased amygdala response to angry or fearful faces^10–13^. This would be the case for NM, since they are more likely to have been neglected or abused in their infancy^5^. However, other studies^14^ did not find such an amygdala response in mothers who have faced early life adversity when viewing emotions other than anger (e.g., sadness or distress). Therefore, it remains unknown whether any of the face-related impairments underlie maternal neglect. Here, we provide evidence for the association between anomalous brain activity in response to crying faces and the maternal neglect of their child.

An intriguing question is to what extent the NM’s presumably altered responses to crying faces are similarly shown in infant and adult faces. While adult and infant crying are powerful signals of conspecific distress^15^, it has been shown that adults, in general, are uniquely attuned to social–emotional signaling from infant faces^16,17^. A number of recent studies have explored the neural basis of processing differences for the infant as compared to adult faces. Using magnetoencephalography in adults, infant faces compared to adult faces triggered fast brain activity in the orbitofrontal cortex, though viewing both adult and infant faces elicited similar activation patterns, which were initiated in the posterior visual cortex and spread along both ventral and dorsal visual pathways^18^. As compared to adult faces, infant faces strongly activate the pre-supplementary motor area, lateral PFC, cingulate cortex, and insula, as well as regions involved in visual attention, such as the fusiform gyrus, in non-parent adults and nulliparous woman^19,20^. Furthermore, emotional infant faces have been found to recruit limbic regions, including the amygdala, as well as multiple areas in the frontal cortex, including the lateral PFC, in nulliparous woman^21^. However, whether infant crying faces evoke differential responses with regard to adult crying faces in NM, remains unknown.

To test for altered face-related activity in NM versus CM, we examined brain responses to (infant and adult) crying faces as compared to neutral faces. We tested whether there are different responses to infant crying faces with respect to adult crying faces, to disentangle what is generic (affecting both types of faces) from what is specific (infant faces affected differently from adult faces). Given that we have manipulated both Group (NM vs CM) and Type of Face (infant and adult crying versus neutral faces), we tested first the extent to which the NM’s alterations were generic, indicated by a main effect of Group. We predicted that the affected areas would be mainly located in the visual-limbic pathway (e.g., lingual, fusiform, amygdala, hippocampus). In favor of this hypothesis, there are empirical grounds to predict that alterations in NM would underlie not only their inadequate caregiving to their child but also their poor abilities exhibited in social encounters in general. NM showed structural anomalies in the ILF connecting temporo-occipital face-responsive areas and the limbic system^8^ that would presumably affect both infant and adult faces. NM self-reports have also shown low empathic concern and high social anhedonia, two dispositional traits that imply a tendency toward emotional avoidance and social disengagement^7^.

Meanwhile, the specificity hypothesis predicting that processing infant faces is affected in different ways from adult faces would be supported if we found a Group by Type of Face interaction. In this case, we predicted that the specific alterations that differentiate NM and CM would be located in regions underlying the parental sensitive responses to infant faces recently highlighted across cultural groups (e.g., anterior insula, supplementary motor area, cingulate cortex, inferior frontal regions)^22–24^. Moreover, we also expected preferential neural processing of infant crying faces in CM but not in NM^10,21^.

Neglectful mothers are more likely than non-neglectful mothers to have mental health problems. A meta-analytic review of risk factors for child neglect revealed that parental variables such as having a history of mental/psychiatric problems and having mental/physical problems are among the most relevant risk factors^25^. Therefore, it is important to take into account such comorbidity when exploring NM / CM brain differences in response to crying faces. Many studies on mood and anxiety disorders showed increased amygdala response to angry or fearful faces^23^, and a few indicated a hyper-activation of the amygdala following exposure to sad expressions in major depression^26–28^. A decreased functional connectivity between amygdala and nucleus accumbens has been also found in maternal depressive mood^29^. Modeling this comorbidity may help to reveal a clearer picture of the neural alterations in NM. In sum, this study aimed to examine for the first time whether processing infant and adult crying faces share a common neural network in both groups or are differentially processed by NM and CM.

## Methods

### Participants

Forty-eight mothers (25 NM and 23 CM) were recruited through the same Primary Health Centers in Tenerife, Spain. The inclusion criterion for NM was having a child under three years old, and that the child had been registered in the last 12 months by Child Protective Services (CPS) as a substantiated case of neglect. CM had a confirmed absence of CPS or Preventive Services records for the family. All mothers in the neglectful group exhibited the three main subtypes of neglect and scored positively on all indicators: physical neglect (inadequate food, hygiene, clothing, and medical care), lack of supervision (child is left alone or in the care of an unreliable caregiver), and educational neglect (lack of cognitive and socioemotional stimulation and lack of attention to child’s education), according to the Maltreatment Classification System^30^. None of the CM scored positively on any of the indicators for the three subtypes of neglect. None of the infants in any group had been placed in foster care at any point in their history, nor had they been born prematurely or suffered perinatal or postnatal medical complications. NM and CM were sociodemographically similar in the age of the target child, living area, education, and unemployment status. They differed slightly in age (on average, CM were three years older, age not being relevant in activation studies -as it would be in volumetric studies), and the number of children (on average, NM had more children, also not relevant since we do not include any primiparous-multiparous contrasts). According to social workers’ reports on the risk profile (presence or absence of indicators), the majority of NM compared to CM had a history of childhood maltreatment or neglect, and the typical risk profile of maternal neglect was also confirmed in the NM group (Table 1; see Supplementary information for more details of the risk profile variables).

**Table 1:** Sociodemographic and neglect risk profiles of mothers in Neglectful and non-neglectful Control groups.

	Neglectful group (^#^*n* = 23) *M* (*SD*) or %	Control group (^#^*n* = 20) *M* (*SD*) or %	*t(41)*/ *χ*^2^
**Sociodemographic profile**			
Mean age of mother	29.1 (7.2)	33.95 (3.1)	−2.99 **
Number of children	2.13 (0.8)	1.5 (0.5)	2.61*
Mean age of the target child	2.6 (1.3)	2.1 (1.7)	1.02
Rural areas (%)	13.0	25.0	0.37
Level of education (%):			3.79
Primary	69.6	40.0	
Secondary school	17.4	35.0	
>Secondary school	13.0	25.0	
Unemployed (%)	87.5	82.6	5.32
**Neglect Risk Profile**			
History abuse/neglect (%)	65.2	10	11.43***
Intimate partner conflict (%)	19	0	1.47
Chronic physical illness (%)	19	0	1.47
Poor household management (%)	88	0	21.46***
Disregard health/education needs (%)	62	0	11.78***
Disregard emotion/cognitive needs (%)	88	0	21.46***
Rigid/inconsistent parental norms (%)	75	0	16.13***

*p ≤ 0.05; **p ≤ 0.01; ***p ≤ 0.001.

^#^These figures correspond to the final sample submitted to the fMRI analyses.

Worse psychiatric and cognitive conditions were found in the neglectful group: psychiatric conditions were measured by the Mini International Neuropsychiatric Interview (Spanish version^31^, and cognitive conditions by the Mini-Mental State Examination (Spanish version^32^, Psychiatric scores were obtained from a cumulative scoring of items and did not correspond to a categorical diagnostic classification (see Supplementary Table S1). The six psychopathological variables that survived Bonferroni correction and that most differentiated between the two groups of mothers were related to mood and anxiety disorders (Major Depressive Disorder, Hypo/Manic Episode, General Panic Disorder, Obsessive-Compulsive Disorder, Generalized Anxiety Disorder) and to antisocial personality, and not to addictions or psychotic conditions. To model the possible influence of psychiatric disorders on the brain responses and to reduce the number of covariates in the SPM analysis, we submitted those variables to a Principal Component Analysis using R toolbox^33^. The results gave a one-factor solution with high inter-correlations among the six factors, KMO = 0.73, Eigenvalue = 3.39, with an explained variance of 86%. The coefficient scores in this factor, named as Psychiatric Disorders (PD), higher in NM (*M* = 0.58, *SD* = 1.04) than in CM (*M* = −0.68, *SD* = 0.2), *t*(23.85) = 5.71; *p* < 0.001; Delta = 1.74, were used as a regressor on the functional imaging data.

### Ethical statement

All methods were carried out in accordance with the Helsinki Declaration. Ethical approval for the study was granted by the Comité de Ética de la Investigación y de Bienestar Animal of the University of La Laguna. All participants in this study provided written informed consent for participation. Mothers who participated in previous studies provided written informed consent for publication of identifying images of infant faces shown in an online open-access publication. Adult faces shown were taken from the Karolinska Directed Emotional Faces Database (KDEF) and reprinted with permission.

### Infant and adult stimuli and paradigm

**Figure 1:**
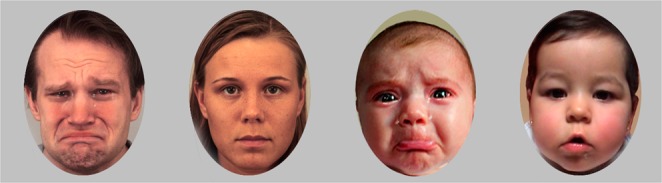
Examples of photographs used as stimuli, representing the four conditions of the study: adult crying and neutral faces (Id numbers BM17SAS and AF19NES from the KDEF database), infant crying and neutral faces (from our own database).

Fifty-six pictures of crying and neutral infant faces (50% male) were taken from our own database of pictures of white children up to three years old provided by mothers from a wider sample of previous studies under written informed consent. Fifty-six pictures of adult sad and neutral faces (50% male) were adapted from the KDEF database^34^ (see identification number of the adult faces used in Supplementary material). The sad faces were edited to add tears and other features to maximize the realism of a crying expression. All images were in color, frontally oriented within an oval frame, and matched for size and brightness using Adobe Photoshop 8.0.1 (see Validation study in Supplementary material). All mothers participated in one 17-minute event-related two-run fMRI experiment, with each run involving all 112 facial expressions, presented randomly. Twenty additional smiling faces (10 infants and 10 adults) from the same databases were presented as catch trials requiring response by moving a lever. The high percentage of accuracy of the mothers included in the fMRI study guarantees their attention to the task [CM (*M* = 96.5; *SD* = 3.75) and NM (*M* = 94.4; *SD* = 13.31)], with no significant differences between groups. All pictures were presented for 1 s. In order to optimize the statistical efficiency of the design, the variable interstimulus interval (ISI) was presented in different (“jittered”) durations across trials (1.87, 3.56, 4.96 s, in the proportion of 57:28:15)^35^. Figure 1 illustrates examples of each category.

### MRI acquisition

Brain images were acquired using a 3T GE Sigma Excite MRI scanner at the University Hospital’s Magnetic Resonance Service for Biomedical Research. The fMRI scan consisted of 287 echoplanar images (T2*-weighted gradient-echo pulse sequence: FOV = 240 mm; TE = 22 ms; TR = 1800 ms; FA = 90°; 33 slices; thickness = 3.5 mm; DF = no gaps; voxel size = 3,75 × 3,75 × 3,5. A T1 anatomical image was also recorded: FOV = 240 mm; TE = 1.736 ms, TR = 8.716 ms, 196 slices, thickness = 1 mm, matrix = 256 × 256 mm^2^).

### FMRI data reduction and analysis

The fMRI data of each individual subject were explored using the Artifact Repair toolbox (Gabrieli Cognitive NeuroScience Lab; http://cibsr.stanford.edu/tools/ArtRepair/ArtRepair.htm). Five mothers had more than 40% of the scan-to-scan motion >1 mm and were therefore excluded. Thus, 23 NM and 20 CM were finally used to estimate the group effects. Functional data were analyzed using SPM8 and related toolboxes (http://www.fil.ion.ucl.ac.uk/spm). Raw functional scans were slice-time corrected taking the middle slice as a reference, spatially realigned, unwarped, coregistered with the anatomical T1, and normalized to the MNI space using the unified normalization segmentation procedure. Normalized images were then smoothed using an isotropic 8 mm Gaussian kernel. The resulting time series from each voxel were high-pass filtered (128 s cut-off period).

Statistical parametric maps were generated by means of the univariate general linear model (GLM), using, for each stimulus type, a regressor obtained by convolving the canonical hemodynamic response function with delta functions at stimulus onsets, and also including the six motion-correction parameters as regressors. The stimulus onsets included six different variables corresponding to each of the four experimental conditions (Infant crying, Infant neutral, Adult crying, Adult neutral), the catch trials, and the fixation baseline. GLM parameters were estimated with a robust regression using weighted-least-squares that also corrected for temporal autocorrelation (http://www.bangor.ac.uk/~pss412/imaging/robustWLS.html). As we were interested in the emotional effects, a pair-wise contrast was performed comparing activation of infant and adult crying conditions relative to their respective neutral conditions (crying > neutral faces; infant and adult crying faces thereof). The resulting images from this first level pair-wise contrast were then used for the second-level analysis.

The statistical parametric maps were then submitted into a 2 × 2 factorial design (i.e., in SPM, Full Factorial Design; using Group (CM vs NM) and Type of Face (Infant vs Adult crying faces) as between-subject and within-subject factors, respectively. The factor Psychiatric Disorders (PD) was also included as a covariate to model its possible effect. Although the dichotomized Group of mothers and PD were certainly related (r = 0.64), to rule out potential collinearity and consequent artifact effects, three indexes of collinearity were calculated using R toolbox^33^: the Variance Inflation Factor, (VIF), the Tolerance (TOL), and the Condition Number (CN). Results showed that all the values fall below the cutoffs for collinearity in the three indexes. Next, we further calculated the index of shared variance between the two variables (Group and PD), with the results showing that the value falls below the allowed threshold (see Supplementary Table S2 for statistical details and references). Once those requirements were satisfied, PD was included as a covariate in the SPM model to control as much as possible for its effect on brain activation differences.

## Results

### Generic deficits in response to crying faces (Main effect of Group)

**Figure 2:**
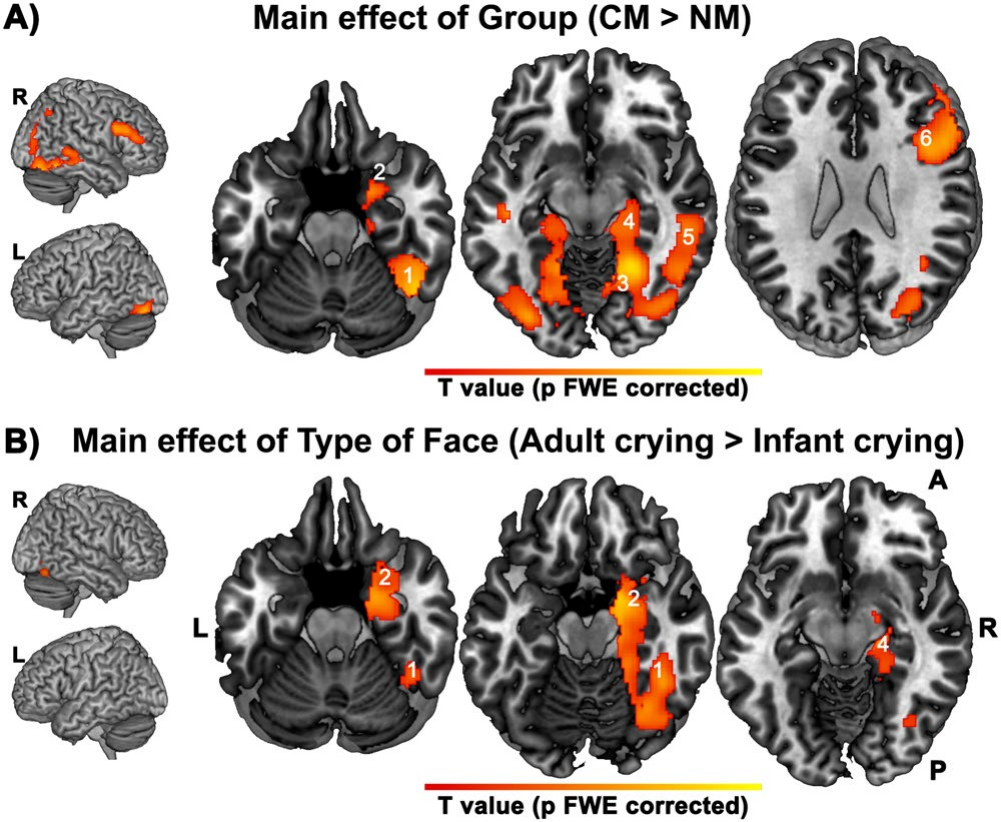
Significant clusters resulting from the contrast Control > Neglectful mothers showing attenuation to crying faces -Generic Hypothesis- (**A**) and clusters resulting from the contrast Adult Crying > Infant Crying Faces (**B**). Panel (A) Neglectful mothers as compared to control mothers show attenuation in Fusiform (1), Amygdala (2), Lingual (3), Hippocampus (4), Inferior Temporal (5), Inferior Frontal Gyrus (6). Neglectful mothers show a higher response to adult crying with respect to infant crying faces in Anterior Cingulate (in green); CM mothers show a higher response to infant crying respect to adult crying faces in left Middle Frontal (in fuchsia). Panel (B) Clusters show increased response to adult crying compared to infant crying in the right vermis, Fusiform (1), Amygdala (2), Hippocampus (4), and Inferior Temporal. All the clusters reported were significant after whole-brain voxel level correction (p-value FWE corrected < 0.05).

Maternal neural responses to crying faces (crying minus neutral) showed a lower response in extended face-processing areas in the NM than in the CM (Table 2). Whole-brain voxel level corrections were applied. All the reported local maxima belong to significant clusters (*p* value FWE corrected < 0.05). The areas affected by decreased responses in NM were bilateral lingual, bilateral cerebellum 6, bilateral fusiform gyrus, right hippocampus, parahippocampal gyrus, and right amygdala. The same pattern (NM < CM) was found in frontal areas involving the Pars Triangularis and Pars Opercularis within the inferior frontal gyrus in response to crying faces. No cluster response was found in the reverse comparison (NM > CM). Figure 2A illustrates the main areas with decreased response in NM as compared to CM.

**Table 2:** Regions activated for the main effects of Group and Type of Face.

Region	x,y,z {mm}		Cluster size (voxels)	Peak T value	
**Main effect of Group (Control Mothers** > **Neglectful Mothers)**					
Fusiform_R	24	−52	−14	3370	4.74
Lingual_R	22	−52	−7	3370	4.73
Temporal_Inf_R	54	−36	−18	3370	4.85
ParaHipocampal_R	22	0	−22	3370	3.79
Cerebelum_6_R	30	−54	−22	3370	3.79
Amygdala_R	25	2	−22	3370	3.61
Hippocampus_R	20	−18	−18	3370	3.39
Fusiform_L	−36	−78	−16	1307	5.15
Lingual_L	−17	−52	−7	1307	3.56
Cerebelum_6_L	−32	−54	−20	1307	4.20
Frontal_Inf_Oper_R	50	14	26	880	4.23
Frontal_Inf_Tri_R	54	26	24	880	4.05
Frontal_Inf_Tri_R	48	36	16	880	3.65
**Main effect of Type of Face (Adult crying** > **Infant crying faces)**					
ParaHippocampal_R	18	0	−18	3325	4.39
Fusiform_R	42	−46	−18	3325	3.73
Hippocampus_R	30	−10	−22	3325	3.39
Amygdala_R	14	−16	−16	3325	4.11
Vermis_6	2	−64	−20	3325	4.32

### Differences between adult and infant faces (Type of face main effect)

Processing adult versus infant crying faces induced a pattern of response in vermis 6, right fusiform, right hippocampus, parahippocampal area, and right amygdala in both groups (Fig. 2B). Whole-brain voxel level corrections were applied. All the reported local maxima belong to significant clusters (*p* value FWE corrected < 0.05).

### Specific deficits (Group x Type of Face interaction)

**Figure 3:**
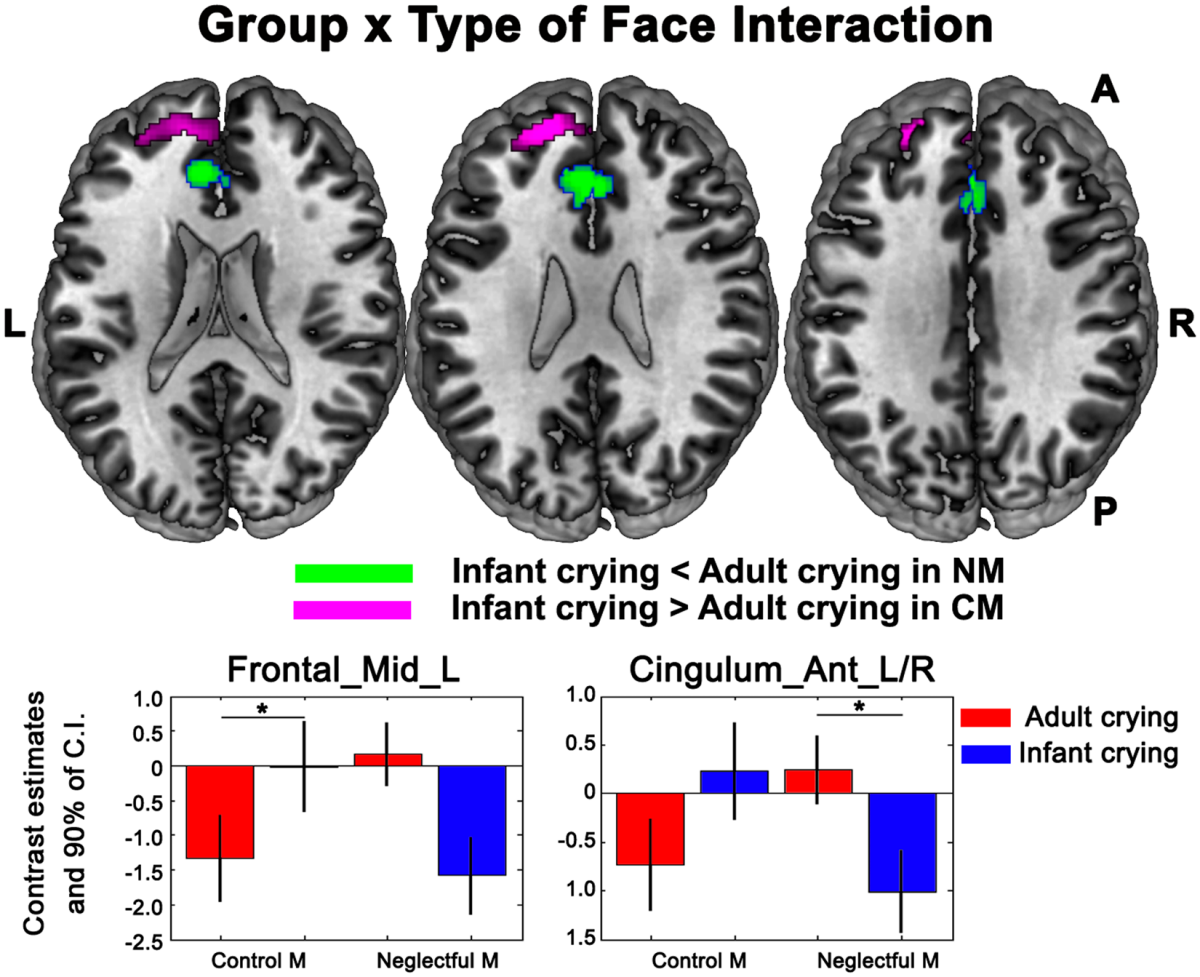
Significant clusters resulting from the Group x Type of Face interaction showing similar pattern of responses for both clusters, but different significant simple effects in CM and NM -Specific Hypothesis-. The middle frontal cluster shows an increased response to infant crying compared to adult crying faces in CM. The anterior cingulate cluster shows an attenuated response to infant crying compared to adult crying faces in NM. The bar graphs display the estimated contrasts (adjusted mean) per condition and 90% of confidence intervals at the maximum peaks representative of the two clusters and indicate with asterisks the two significant comparisons. MANOVA results show different significant simple effects for infant and adult crying faces in NM and CM. All the clusters reported were significant after whole-brain cluster level correction (p-value FWE corrected < 0.05).

We searched for more specific deficits for each type of face separately in NM as compared to CM, testing the Group x Type of Face interaction. Whole-brain cluster level corrections were applied. All the reported local maxima belong to significant clusters (*p* value FWE corrected < 0.05). This analysis showed that the two groups of mothers process infant and adult crying faces differently. Specifically, we observed significant Group x Type of Face interaction effects in two left frontal clusters that included the middle and superior frontal area, the superior medial frontal area and in two cingulate cortex clusters (anterior and middle) (see Table 3 for more details). The post-hoc comparisons that followed from the interaction resulted in two significant simple effects in two clusters: bilateral anterior cingulate and left middle frontal. Although in both clusters, the direction of the response to infant and adult faces is the same, only one simple effect in each cluster was significant for each group of mothers: A higher response for the infant as compared to adult crying faces in the frontal middle area for the CM; and a lower response for infant as compared to adult crying faces in the anterior cingulate cortex for the NM (Fig. 3).

**Table 3:** Regions belonging to the two clusters activated for the Group x Type of Face interaction and the posthoc significant comparisons.

Region	x,y,z {mm}		Cluster size (voxels)	Peak T value	
**Group x Type of Face Interaction**					
Frontal_Mid_L	−28	50	22	323	4.01
Frontal_Sup_L	−16	56	26	323	3.25
Frontal_Sup_Med_L	−4	54	22	323	3.00
Cingulum_Ant_L/R	−8	38	24	490	3.79
Cingulum_Mid_L	0	24	36	490	3.14
**Simple effect - Infant** > **Adult crying faces in Control Mothers**					
Frontal_Mid_L	−16	54	26	213	4.13
**Simple effect - Adult** > **Infant crying faces in Neglectful Mothers**					
Cingulum_Ant_L/R	−8	36	24	262	3.31

## Discussion

We provide the first evidence that NM present a reduced face-related response to infant and adult crying faces in extensive areas of the visual-limbic pathway that subserves emotional, learning, and memory functions, with extension to specific frontal regions. We confirm the generic hypothesis predicting altered neural processing in NM of both infant and adult crying faces, in contrast to neutral faces. A reduced response in NM was found in the cerebellum, recently claimed to be involved in emotional face processing^36^, and in the visual cortex (bilateral lingual and fusiform), all of which were also found in mothers of infants displaying insecure attachment^37^. The reduced cerebellum response, as well as the right hippocampus and parahippocampus attenuation, is not surprising if we consider that all of these areas are vulnerable to stress due to their high level of glucocorticoid receptors^38,39^, which is more likely to be the case for NM who exhibited a more severe stress risk profile than CM. Also, NM showed a reduced response of the right amygdala, a key region which monitors the affective relevance of incoming stimuli^40^ and is related to both maternal affect^41^ and to maternal personal distress when receiving negative feedback (a child’s unhappy face) in a caregiving task^42^. A reduced response in NM was also found in areas of the mirror neuron system^43^, including the empathy-related area involved in sympathizing with others’ distress, such as the right inferior frontal gyrus^44^. As for the Type of faces main effect, we found an increased response in both groups of mothers to adult crying faces as compared to infant crying faces in the right hemisphere of the visual-limbic pathway. Probably, seeing the disturbing adult expressions as a signal of heavy distress^15^ triggered an enhancement of the perceptual, emotional, and memory processing.

As neglectful mothering involves under-responsiveness to infants, infant crying faces are particularly relevant stimuli to be examined in this study. In favor of the specific alteration hypothesis, we found an interaction pattern of responses to infant crying versus adult crying faces that differed across the two groups. As expected, the areas that discriminate between infant and adult crying faces comprised medial and frontal areas, such as bilateral ACC and MCC, as well as middle and medial PFC^22,23^, which underlie the parental sensitive responses to infant crying faces relative to adult crying faces^19–21^. We also obtained a clear-cut picture of the direction of the interaction results. Thus, infant crying faces as compared to adult faces, recruited a higher response of the middle frontal area, confirming its preferential processing in CM^19,21^. In turn, infant crying faces as compared to adult faces, recruited in NM a lower response of the anterior cingulate cortex, a structure involved in parental sensitive responses to infant crying faces^22,23^.

As expected, NM have more mental health problems than CM, which are among the major risk factors for child neglect^25^. For this reason, we also modelled the effect of psychiatric disorder comorbidity on the pattern of brain response. We obtained an atypical pattern consisting of the lowered response of the amygdala in NM to crying faces that differs from the amygdala hyper-reactivity found in response to angry and fearful faces associated with early life adversity^10–13^, and to sad faces in major depression. However, some studies with mothers and children with early life adversity and/or psychopathological risk have obtained opposite effects. Traumatized mothers showed reduced bilateral amygdala response when viewing their own infants’ distress as compared to their happiness^14^. Children exposed to domestic violence did not show an increased response in the amygdala and anterior insula when viewing sad faces, but just in response to angry faces^45^. In the same vein, children of mothers with a history of major depression during their children’s lives exhibited greater attentional avoidance of sad faces but not of happy or angry faces^46^. We argue that the reduced amygdala response found in neglectful mothers may be adaptive since it provides a short-term functional advantage by promoting emotional avoidance in their early adverse environment. However, when they become mothers, these neural modifications associated with poor psychiatric conditions may lead to a lack of sensitivity to others’ distress (i.e., their own child’s distress signals) and also to social disengagement during adult exchanges, as shown by their low empathy and high anhedonic tone^7^.

In spite of the robust results showing the first evidence of the differential brain response associated with NM, the composition of the sample in this study did not permit separating out the contribution of the psychiatric conditions to negligent motherhood. Building on the neural differences found in this study, future research, with larger samples, would allow for an orthogonal design crossing NM and CM with other risk factors (i.e., psychopathological conditions, own childhood maltreatment or epigenetic factors) to determine their respective contribution to the neural alterations associated with maternal neglect.

In conclusion, our findings add significantly to the study of neural processing of infant crying and adult faces in atypical cases of motherhood. First, the attenuated brain response to infant and adult crying faces in the limbic-visual circuit pointed to a generic deficit in NM that may affect not only their caregiving role but also their ability to effectively deal with adult social relationships. The general attenuation of the visual-limbic system is indicative that essential components of the emotional processing system – specifically those subserving the automatic neural identification of a stimulus as relevant, the allocated attention to the stimulus, and action readiness to respond to it – are failing in neglectful mothering. Second, the pattern of specific alterations in frontal and anterior cingulate areas indicated that infant and adult crying faces are differentially processed by NM and CM. Enhanced sensitivity to infant faces as compared to adult faces was found in frontal areas in CM, typically involved in maternal sensitive responses^9,22,23^. In turn, the reduced response to infant faces in the anterior cingulate area in NM, also involved in parental sensitivity, goes in the direction that this alteration may be contributing to maternal disregard of the infant’s needs. Put together, these findings support the importance of developing more focused intervention strategies, targeting the altered neurological responses, based on training the mothers in the adequate interpretation of and response to infant signals, as well as to improve their abilities in social relationships as both, directly and indirectly, affect their caregiving role.

## Supplementary information

Limbic-visual attenuation to crying faces underlies neglectful mothering

## Data Availability

The functional and structural MRI data and the covariate scores that support the findings of this study are available in GIN: https://doid.gin.g-node.org/2bf64c7c618df0ef08cfb3f87406f277/, with the identifier, 10.12751/g-node.2bf64c.
