# Supplementary material for: Limbic-visual attenuation to crying faces underlies neglectful mothering
Source: Sci Rep. 2019 Apr 23;9:6373. doi: 10.1038/s41598-019-42908-1 (PMC6476884; doi:10.1038/s41598-019-42908-1)
Supplement: Supplementary file 1 — Limbic-visual attenuation to crying faces underlies neglectful mothering [file 41598_2019_42908_MOESM1_ESM.pdf]

**SUPPLEMENTARY INFORMATION**

**Limbic-visual attenuation to crying faces underlies neglectful mothering**

**Inmaculada León, María José Rodrigo, Wael El-Deredy, Cristián Modroño, Juan Andrés Hernández-Cabrera, and Ileana Quiñones**

## Method

**Table S1.** Psychopathological and Cognitive conditions stratified by Group

| | Neglectful group ($n$ = 23) $M$ ($SD$) | Control group ($n$ = 20) $M$ ($SD$) | $t$(41) | Effect size δ |
|---|---|---|---|---|
| Major Depressive Episode | 2.17 (2.6) | 0.25 (0.55) | 3.19** | 0.98 |
| Dysthymia | 1.43 (1.86) | 0.26 (0.56) | 2.62 * | 0.80 |
| Suicidality | 0.62 (1.1) | 0 (0) | 2.52* | 0.77 |
| Hypo/Manic Episode | 2.05 (2.2) | 0.11 (0.32) | 3.73*** | 1.14 |
| General Panic Disorder | 7.04 (5.87) | 0.84 (2.31) | 4.34*** | 1.36 |
| Agoraphobia | 0.76 (0.94) | 0.26 (0.65) | 1.92 | 0.59 |
| Social Phobia | 0.76 (1.22) | 0 (0) | 2.71** | 0.83 |
| Obsessive-Compulsive | 1.31 (1.70) | 0 (0) | 3.08** | 0.94 |
| Post-traumatic Stress Disorder | 1.12 (1.26) | 0.50 (0.63) | 1.77 | 0.54 |
| Alcohol Dependence/Abuse | 0.06 (0.25) | 0.12 (0.50) | -0.45 | 0.14 |
| Drug Dependence/Abuse | 0.19 (0.40) | 0 (0) | 1.86 | 0.57 |
| Psychotic Disorders | 0.69 (1.40) | 0.12 (0.50) | 1.51 | 0.46 |
| Anorexia Nervosa | 0 | 0 | - | - |
| Bulimia Nervosa | 0 | 0 | - | - |
| Generalized Anxiety Disorder | 3.52 (3.50) | 0.53 (0.90) | 3.60*** | 1.1 |
| Antisocial Personality | 1.43 (1.25) | 0.16 (0.37) | 4.26*** | 1.30 |
| Cognitive Integrity | 25.96 (1.97) | 28.70 (1.42) | -5.17*** | 1.58 |

*$p \leq .05$; **$p \leq .01$; ***$p \leq .001$

**Identification number of the adult faces taken from the Karolinska Directed Emotional Faces Database (KDEF).**

Man sadness (edited as crying faces):
AM05SAS, AM08SAS, AM11SAS, AM12SAS, AM17SAS, AM18SAS, AM19SAS, AM23SAS, AM25SAS, AM28SAS, AM32SAS, AM34SAS, AM35SAS, AM18SAS.

Man neutral:

AM01NES, AM03NES, AM04NES, AM07NES, AM09NES, AM010NES, AM013NES, AM014NES, AM021NES, AM022NES, AM024NES, AM026NES, AM027NES, AM028NES.

Woman sadness (edited as crying faces):
AF01SAS, AF02SAS, AF03SAS, AF06SAS, AF07SA, AF09SAS, AF15SAS, AF17SAS, AF20SAS, AF25SAS, AF29SAS, AF30SAS, AF33SAS, AF35SAS.

Woman neutral:
AF04NES, AF10NES, AF12NES, AF14NES, AF16NES, AF18NES, AF19NES, AF21NES, AF22NES, AF23NES, AF24NES, AF27NES, AF32NES, AF34NES.

**Neglect risk profile variables**

Social workers reported on a series of risk indicators (presence: 1; absence: 0), which are commonly used to assess maternal neglect (Department of Health and Human Services, 2015; Petersen, Joseph, & Feit, 2013). History of abuse/neglect refers to whether mothers have been suffering childhood maltreatment (either abuse or neglect) in their own history (scoring 1); Intimate partner conflict refers to whether mothers are experiencing overt conflictive relationships with their partner (scoring 1); Chronic physical illness refers to whether they are currently experiencing poor health conditions permanently or very frequently (scoring 1); Poor household management refers to whether the home is dirty, untidy, irregular meals and dirty clothing (two is enough for scoring 1); Disregard health/education needs refers to lack of or discontinuous medical checks, irregular vaccines, poor support for learning (two is enough for scoring 1); Disregard emotional/cognitive needs refers to poor attention to the child's emotional expressions, and lack of response to infant curiosity (one is enough for scoring 1); Rigid/inconsistent parental norms refers to an application of rules without taking into account the childrearing situations, and arbitrary change of norms applied to the same situations (one is enough for scoring 1).

**Validation study of face stimuli**

A validation study with 38 university students viewing the edited adult crying faces (neutral faces were not changed) and infant faces (crying and neutral) for 1 sec each, showed that participants accurately classified 95% adult crying faces according to the intended expression (representativeness ratings on a 1-7 scale: M = 5.34; SD = 1.04), 95% infant crying (M = 6.01; SD= 0.72), and 74% infant neutral faces (M= 5.19; SD = 0.82). The neutral adult faces belong to the Karolinska Institutet, KDEF database.

**Test of collinearity for the SPM model**

The dichotomized group variable correlates 0.64 with "Psychiatric Disorders" (PD) as a factor score. Such correlation could suggest a high collinearity between the two variables, preventing the use of PD as a covariate in the SPM. However, research shows that the magnitude of such correlation is not a reliable indicator that there are problems

with collinearity between two variables (Belsley, 1991). To this end, we assessed the potential colinearity (CL) calculating three well-known indexes of CL: the Variance Inflation Factor (VIF), the Tolerance (TOL), and the Condition Number (CN). We calculated them for the overall factor PD with the Group, and separately for each psychiatric disorder that survived to Bonferroni test in the group comparisons (Table S2). We also measured the shared variance of the psychiatric variables with Group (last column).

**Table S2.** Collinearity indexes between the Group (as a dichotomic variable) and the psychopathological conditions, both as a factor PD and the individual disorders separately (within brackets are the cutoff values for non-collinearity).

| | VIF (<10) | TOL (>0.30) | CN (<10) | Shared Variance (<0.50) |
|---|---|---|---|---|
| **Factor score "Psychiatric disorder"** | **1.74** | **0.57** | **3.54** | **0.41** |
| Major Depressive Disorder | 1.25 | 0.80 | 1.62 | 0.20 |
| Hypo/Manic Episode | 1.33 | 0.75 | 0.72 | 0.25 |
| General Panic Disorder | 1.48 | 0.68 | 1.90 | 0.32 |
| Obsessive-Compulsive Disorder | 1.22 | 0.82 | 2.07 | 0.18 |
| Generalized Anxiety Disorder | 1.30 | 0.77 | 1.68 | 0.23 |
| Antisocial Personality | 1.38 | 0.72 | 1.79 | 0.27 |

Note: VIF: Variance Inflation Factor, TOL: Tolerance, CN: Condition Number.

According to the literature, the general criteria for non-collinearity is a Variance Inflation Factor (VIF) less than 10, a Tolerance higher than 0.30, a Condition Number less than 10, and a shared variance less than 0.50 (Belsley, 1991; Belsley et al., 2005; Kovács et al., 2005; Tabachnick & Fidell, 2007). Our results (see Table S2) showed that all the values fall below the corresponding cutoffs for collinearity. That made possible the inclusion of the PD as a covariate together with Group in the SPM model, to control as much as possible, its effect on the activation results.

References

Belsley, D. A. (1991). A guide to using the collinearity diagnostics. *Computer Science in Economics and Management*, *4*(1), 33-50.

Belsley, D. A., Kuh, E., & Welsch, R. E. (2005). *Regression diagnostics: Identifying influential data and sources of collinearity* (Vol. 571). John Wiley & Sons.

Department of Health and Human Services, Administration on Children, Youth and Families & Children's Bureau Services & Washington, DC (U.S): Government Printing Office. *Child maltreatment 2013* (2015).

Petersen, A., Joseph, J., & Feit, M. (2013). New directions in child abuse and neglect research. *Report of the Committee on Child Maltreatment Research, Policy, and Practice for the Next Decade: Phase II*.

Kovàcs, P., Petres, T., & Tóth, L. (2005). A new measure of multicollinearity in linear regression models. *International Statistical Review*, 73(3), 405-412.

Tabachnick, B.G. y Fidell, L.S. (2007). *Using Multivariate Statistics* (Quinta Edición). Boston: Pearson Education, Inc.
